# Circ-CSNK1G1 promotes cell proliferation, migration, invasion and glycolysis metabolism during triple-negative breast cancer progression by modulating the miR-28-5p/LDHA pathway

**DOI:** 10.1186/s12958-022-00998-z

**Published:** 2022-09-15

**Authors:** Xiaochen Zan, Wenfang Li, Geng Wang, Jie Yuan, Yongbiao Ai, Jun Huang, Zhi Li

**Affiliations:** Department of General Surgery, Taihe Hospital, Huibei University of Medicine, No. 32th, South Renmin Road, 442000 Shiyan City, Hubei Province PR China

**Keywords:** circ-CSNK1G1, Triple-negative breast cancer, miR-28-5p, LDHA

## Abstract

**Background:**

Circular RNAs (circRNAs) play a vital role in cancer progression. However, there are still numerous circRNAs that have not been functionally explored. Our study aimed to disclose the role of circ-CSNK1G1 in triple-negative breast cancer (TNBC).

**Methods:**

The expression of circ-CSNK1G1, miR-28-5p and lactate dehydrogenase A (LDHA) mRNA was measured by quantitative real-time polymerase chain reaction (qPCR), and the expression of LDHA protein was measured by western blot. Cell proliferation was assessed using MTT assay and colony formation assay. Cell apoptosis was monitored using flow cytometry assay. Cell migration and cell invasion were investigated using transwell assay. Glycolysis progression was assessed according to glucose consumption, lactate production and ATP/ADP ratio. Tumor formation assay in nude mice was conducted to verify the role of circ-CSNK1G1 in vivo. The interplays between miR-28-5p and circ-CSNK1G1 or LDHA were confirmed by dual-luciferase reporter assay.

**Results:**

Circ-CSNK1G1 was upregulated in TNBC tissues and cells. Circ-CSNK1G1 knockdown suppressed cancer cell proliferation, migration, invasion and glycolysis energy metabolism, promoted cell apoptosis in vitro, and blocked tumor growth in vivo. Mechanism analysis showed that circ-CSNK1G1 positively regulated LDHA expression by suppressing miR-28-5p. Rescue experiments presented that circ-CSNK1G1 played functions by targeting miR-28-5p, and miR-28-5p participated in TNBC progression by degrading LDHA.

**Conclusion:**

Circ-CSNK1G1 promotes cell proliferation, migration, invasion and glycolysis metabolism during TNBC development by regulating the miR-28-5p/LDHA pathway.

**Supplementary Information:**

The online version contains supplementary material available at 10.1186/s12958-022-00998-z.

## Highlights

1. Circ-CSNK1G1 is upregulated in TNBC.

2. Circ-CSNK1G1 knockdown inhibits the proliferation, migration, invasion and glycolysis energy metabolism of TNBC cells.

3. Circ-CSNK1G1 knockdown inhibits tumor formation in vivo.

4. Circ-CSNK1G1 promotes TNBC development by regulating the miR-28-5p/LDHA pathway.

**Supplementary Information:**

The online version contains supplementary material available at 10.1186/s12958-022-00998-z.

## Background

There were approximately 62,930 new cases of female breast cancer in the USA in 2019, and breast cancer alone occupies 30% of all newly diagnosed cancers in women [1]. Though the mortality of patients with breast cancer had declined in recent years, a third of breast cancer sufferers develop distant metastases and eventually die from the disease [2]. Triple-negative breast cancer (TNBC) is a rare subtype of breast cancer, with high invasiveness, poor prognosis and high mortality [3]. The current research focuses on biomarkers, such as circulating tumor DNA (ctDNA), RNA (such as non-coding RNAs), circulating tumor cells, and peroxisome proliferator-activated receptor [4–8]. More risk factors contributing to TNBC should be identified to provide additional therapy opinions for this disorder.

It is well known that circular RNA (circRNA) is one kind of non-coding RNAs. Numerous circRNAs have been functionally addressed in TNBC, such as circZEB1, circPGAP3 and circAGFG1 [9–11], which provides new insights into the understanding of mechanisms of tumor cell proliferation and metastasis. CircRNAs are generated from precursor mRNAs via alternative splicing, termed as back-splicing [12]. Due to the advance of RNA sequencing technology, a growing number of circRNAs have been shown to be cell- and tissue-specific in cancer [12]. The molecular functions of circRNA are multi-faceted, acting as microRNA (miRNA) decoys, transcriptional regulators and protein-like modulators [13]. Circular casein kinase 1 gamma 1 (circCSNK1G1; circ_0001955) is derived from exon5-exon10 of CSNK1G1 mRNA. A circRNA profile (GEO accession: GSE101123) recorded differently expressed circRNAs in breast cancer [14], and we analyzed the dataset and found that circCSNK1G1 was highly expressed in breast cancer. Nevertheless, the function of circCSNK1G1 is rarely investigated in TNBC until now.

When it comes to circRNAs, miRNAs cannot be ignored because it is canonical that cytoplasmic circRNAs function as miRNA sponges [15]. MiRNAs govern multiple biological functions by regulating the expression of target genes via binding to their 3’ untranslated region (3’UTR) [16]. The analyses from the online bioinformatics tool starbase hint that circCSNK1G1 harbors miR-28-5p binding sites, and miR-28-5p binds to lactate dehydrogenase A (LDHA) 3’UTR. Previous studies have proposed that miR-28-5p and LDHA may be involved in breast cancer development [17, 18]. Therefore, it is reasonably speculated that miR-28-5p associated with LDHA may be involved in the regulatory network governed by circCSNK1G1.

In the current study, we obtained circCSNK1G1 from a previous circRNA profile. We examined the expression of circCSNK1G1 in clinical tumor tissues and cell lines of TNBC and next investigated the functions of circCSNK1G1 in TNBC cells in vitro and in solid tumor growth in vivo. Besides, we mechanically explored the interplays among circCSNK1G1, miR-28-5p and LDHA. Our study aimed to determine the role of circCSNK1G1 and further understand the pathogenesis of TNBC.

## Materials and methods

### Patients and samples

Patients with TNBC (*N* = 33) were recruited from Taihe Hospital, Huibei University of Medicine. Tumor tissues and non-cancer tissues were surgically excised from patients with written informed consent. None of the patients had received any radiotherapy and chemotherapy before surgery. Samples were frozen in liquid nitrogen and preserved at -80℃ conditions. This study obtained the permission of the Ethics Committee of Taihe Hospital, Huibei University of Medicine.

### Cell lines

All used cell lines were purchased from Bena culture collection (Beijing, China) and maintained at 37℃ conditions supplemented with 5% CO_2_. According to the instructions, MCF-10 A (non-cancer cells; control) and MDA-MB-231 cells were cultured in 90% DMEM (Bena) containing 10% FBS (Bena). MDA-MB-453 and MDA-MB-468 cells were cultured in Leibovitz’s L-15 medium (Bena) containing 10% FBS. BT-549 cells were cultured in RPMI1640 medium (Bena) containing 10% FBS.

### Quantitative real-time polymerase chain reaction (qPCR)

Trizol reagent (Invitrogen, Carlsbad, CA, USA) was used to isolate total RNA from tissues and cells according to manufacturer’s protocol. RNA samples were reverse-transcribed into cDNA using the PrimeScript RT reagent kit (Takara, Dalian, China) or using the TaqMan MicroRNA Reverse Transcription kit (Applied Biosystems, Foster City, CA, USA). Then cDNA was utilized for qPCR using the SYBR Green Master PCR mix (Applied Biosystems) on ABI 7900 system (Applied Biosystems). The expression levels were normalized by GAPDH or U6, using the 2^−ΔΔCt^ method. All procedures were performed following the manufacturer’s instructions. The primers used were displayed in Table 1.

**Table 1:** Primers sequences used in qPCR

Name		Primer sequences (5’-3’)
circ-CSNK1G1	Forward	GGACCCTCTTCACAGACCTC
	Reverse	GGAGACCTTCACCTGATTTCG
CSNK1G1	Forward	TGTATCATCAGAGCGCCGAG
	Reverse	TGAGCTAACCACCTGCACTG
LDHA	Forward Reverse	TTGTCTCTGGCAAAGTGGAT
		ACCGCTTCCAATAACACGGT
miR-28-5p	Forward	GCGCATTGCACTTGTCTCG
	Reverse	AGTGCAGGGTCCGAGGTATT
U6	Forward	GCTTCGGCAGCACATATACTAAAT
	Reverse	CGCTTCACGAATTTGCGTGTCAT
GAPDH	Forward	GAAGGTGAAGGTCGGAGTC
	Reverse	GAAGATGGTGATGGGATTTC

### RNase R digestion

Partial isolated RNA samples were exposed to 1 µg RNase R (2 U/µg, Epicentre, Madison, WI, USA), followed by 1-h incubation at 37℃ conditions. Then, RNA samples were used for qPCR analysis.

### The distribution of circ-CSNK1G1

The Cytoplasmic & Nuclear RNA Purification Kit (Norgen Biotek Corp, Thorold, Canada) was applied to isolated cytoplasmic RNA and nuclear RNA, followed by the quantification of circ-CSNK1G1 using qPCR. GAPDH or U6 was used as the internal reference in the cytoplasmic fraction and nuclear fraction, respectively.

### Cell transfection

For circ-CSNK1G1 silence, small interference RNA (siRNA) specific to circ-CSNK1G1 (si-circ-CSNK1G1: 5’-TTCGAAATCAGGTGAAGGT-3’) and siRNA negative control (si-NC) were synthesized by Geneseed (Guangzhou, China). For circ-CSNK1G1 overexpression, pCD-ciR-circ-CSNK1G1 recombinant vector (circ-CSNK1G1) and empty vector control were constructed by Geneseed. The mimics and inhibitors targeting miR-28-5p (miR-28-5p and anti-miR-28-5p) and their negative control (miR-NC and anti-miR-NC) were purchased from RIBOBIO (Guangzhou, China). For LDHA overexpression, pcDNA-LDHA recombinant vector (LDHA) and empty vector control were assembled by Genecreate (Wuhan, China). All transfections were performed using Lipofectamine 3000 (Invitrogen).

### MTT assay

Cell proliferation was assessed using MTT assay. Cells with different transfection were planted into a 96-well plate (3 × 10^3^ cells/well). After culturing for 24 h, 48 h, and 72 h, cells in each well were treated with 10 µL MTT reagent (Abcam, Cambridge, MA, USA) for 4 h. Dimethyl Sulfoxide (DMSO) was then added to dissolve formazan crystals. Finally, the absorbance values at 490 nm were measured by a microplate reader (Bio-Rad, Hercules, CA, USA).

### Colony formation assay

Colony formation assay was also applied to assess cell proliferation. Cells with different transfection were planted into a 6-well plate (200 cells/well). Cells were next incubated at 37℃ conditions containing 5% CO_2_ for 12 days to induce colony growth. Finally, cell surface was washed with PBS, fixed with paraformaldehyde and stained with crystal violet (Beyotime, Shanghai, China).

### Flow cytometry assay

Cell apoptosis was distinguished by flow cytometry assay. The FITC Annexin V Apoptosis Detection Kit (Invitrogen) was applied for apoptosis analysis in this study. In brief, cells with different transfection were collected after digestion at 48 h post-transfection. Cells were washed with PBS and then treated with 5 µL Annexin V-FITC buffer and 10 µL propidium iodide (PI) solution, for 15 min in the dark. The apoptotic cells (Annexin V-FITC + and PI+/-) were distinguished using a flow cytometer (BD Biosciences, San Jose, CA, USA).

### Transwell assay

Cell migration and cell invasion were determined using transwell assay. Cells with different transfection were collected and resuspended into serum-free culture medium. Cells in serum-free culture medium were placed into the upper of transwell chambers (Corning Incorporated, Corning, NY, USA) coated with or without Matrigel (Corning Incorporated) for invasion assay or migration assay, respectively. The bottom of transwell chambers was filled with culture medium added with 10% FBS. Through 24-h incubation, cells that migrated or invaded into the lower surface were fixed with paraformaldehyde and stained with crystal violet, followed by photograph using a microscope (× 100 magnification; Olympus, Tokyo, Japan).

### 2-DeoxyGlucose (2-DG) treatment

2-DG is an inhibitor of the glycolysis pathway. Cells with different transfection seeded into a 96-well plate were incubated with 50 nM 2-DG. After culturing for 24 h, 48 h, or 72 h, cell viability was checked using by a microplate reader (Bio-Rad).

### Glycolysis analysis

Glycolysis energy metabolism was assessed according to glucose consumption, lactate production and the ration of ATP/ADP. These indexes could be detected using matched kits, including Glucose Assay kit (Abcam), Lactate Assay kit (Abcam) and ADP/ATP Ratio Assay kit (Abcam). Following the protocols, glucose consumption, lactate production and ATP/ADP ratios were investigated to monitor glycolysis progression.

### Tumor formation *in vivo*

For stable circ-CSNK1G1 knockdown, short hairpin RNA (shRNA) targeting circ-CSNK1G1 was synthesized by Geneseed and assembled into a lentiviral vector. BT-549 cells were infected with lentiviral sh-circ-CSNK1G1 (5’-GAAATCAGGTGAAGGTCTCCCTTCAAGAGAGGGAGACCTTCACCTGATTTCTTTTTT-3’) or negative control (sh-NC) and used for tumor formation assay. The experimental mice (Balb/c, female, 6-7-week-old) were purchased from Charles River (Beijing, China). BT-549 cells infected with sh-circ-CSNK1G1 or sh-NC were subcutaneously injected into the groin of mice (*n* = 6 per group). Mice were kept for 8 days until tumor stable growth. Then, tumor volume (length×width^2^ × 0.5) was measured every 3 days. At 23 days post-injection, all mice with anesthesia were sacrificed to remove tumor tissues. Animal study obtained the permission of the Animal Care and Use Committee of Taihe Hospital, Huibei University of Medicine.

### Immunohistochemical (IHC) assay

Paraffin-embedded tissue Sect. (4 µm thick) were prepared. Tissue sections were deparaffinized and dehydrated. After antigen retrieval, sections were incubated with the primary antibodies, including anti-Ki67 (ab92742; Abcam), anti-MMP2 (ab235167; Abcam) and anti-MMP9 (ab228402; Abcam). Tissue sections were next exposed to Goat Anti-Rabbit secondary antibody. The sections were stained with 3, 3’-diaminobenzidine (DAB; Abcam), and then images were taken using a light microscope (Olympus).

### Dual-luciferase reporter assay

The binding sites between miR-28-5p and circ-CSNK1G1 or LDHA 3’UTR were analyzed by starbase v3.0 (http://starbase.sysu.edu.cn/). The wild-type (WT) and mutant (MUT) sequences of circ-CSNK1G1 or LDHA 3’UTR containing miR-28-5p binding sites were inserted into the downstream of dual-luciferase reporter vector PGL4 to forming recombinant vector, WT-circ-CSNK1G1, MUT-circ-CSNK1G1, LDHA 3’UTR-WT and LDHA 3’UTR-MUT. For luciferase assay, MDA-MB-231 and BT-549 cells were cotransfected with miR-28-5p or miR-NC and above mentioned recombinant vector, respectively. After incubating for 48 h, luciferase activity was detected using dual-luciferase reporter assay system (Promega, Madison, WI, USA).

### Western blot

The expression of LDHA protein was detected by western blot. Total proteins were extracted using RIPA lysis buffer (Beyotime) and separated by 12% SDS-PAGE. The separated proteins were transferred onto PVDF membranes, followed by blockage in skim milk. The membranes were incubated with the primary antibodies against LDHA (ab125683; Abcam) and GAPDH (ab9485; Abcam), followed by incubation with the secondary antibody (ab205718; Abcam). Finally, the enhanced chemiluminescence (ECL) (Beyotime) reagent was used to visualize the signal on the membrane.

### Statistical analysis

Statistical analysis was performed using GraphPad Prism 7.0 (GraphPad, Inc., La Jolla, CA, USA). The data were presented as mean ± standard deviation (SD). Student’s t-test and analysis of variance (ANOVA) were applied for difference comparison between two groups and in multiple groups, respectively. The correlation curve was depicted according to Pearson correlation coefficient. The curve of overall survival was generated by Kaplan-Meier plot and analyzed by log-rank test. *P* < 0.05 was considered to be statistically significant. Experiments, including qPCR, MTT assay, colony formation, flow cytometry assay, transwell assay and western blot, were repeated at least three times.

## Results

### Circ-CSNK1G1 was overexpressed in TNBC tissues and cells

**Fig. 1:**
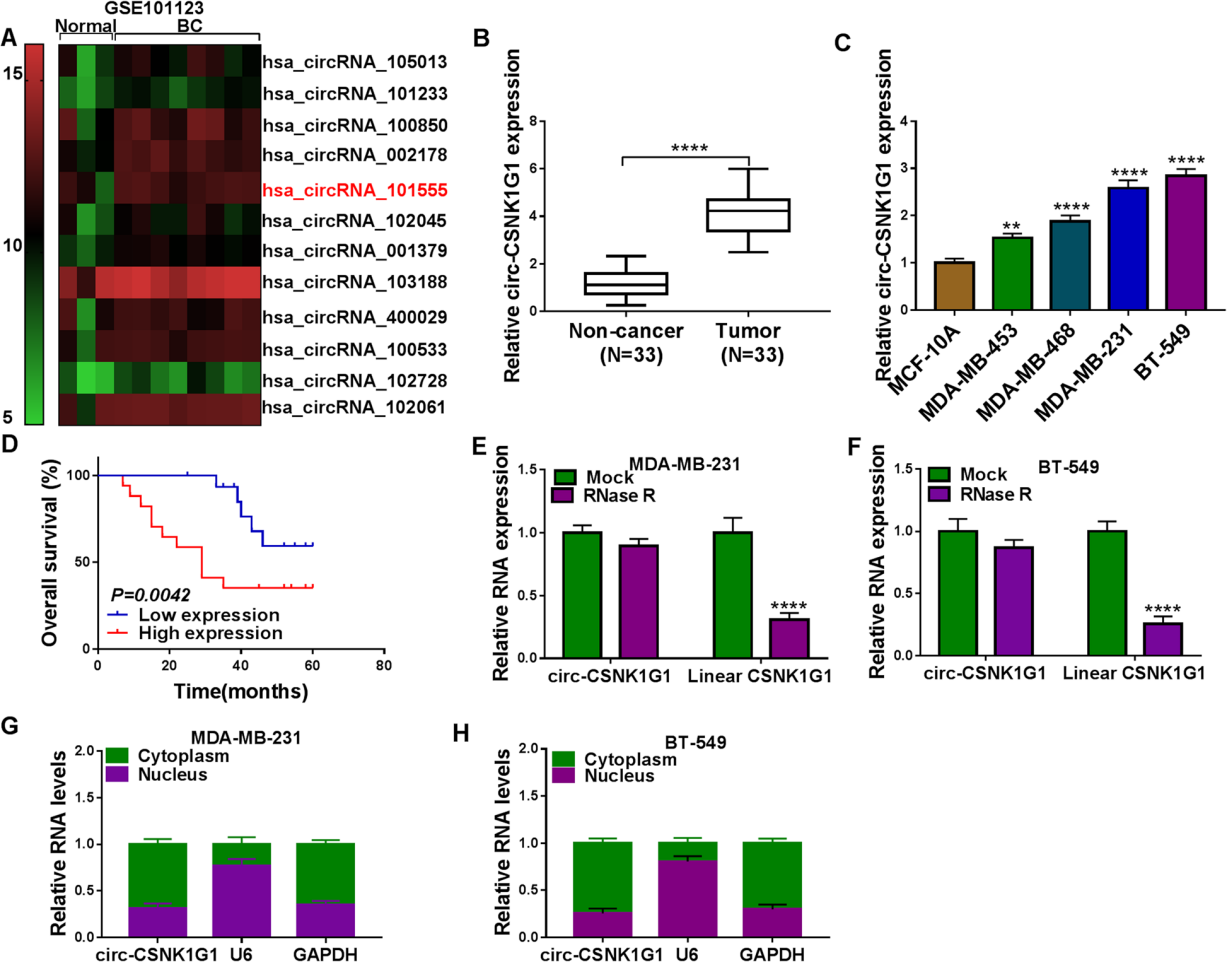
Circ-CSNK1G1 was upregulated in TNBC tissues and cells. **A** The data of circRNA expression in TNBC tissues were collected from a circRNA expression profile (GEO accession: GSE101123). **B** The expression of circ-CSNK1G1 in tumor tissues and normal breast tissues was detected by qPCR. **C** The expression of circ-CSNK1G1 in TNBC cell lines and non-cancer cell line was detected by qPCR. **D** Overall survival was analyzed by Kaplan-Meier plot and log-rank test. **E** and **F** The stability of circ-CSNK1G1 was ensured using RNase R test. **G** and **H** The location of circ-CSNK1G1 was analyzed by qPCR. **P < 0.01 and ****P < 0.0001

A circRNA expression profile (GEO accession: GSE101123) provided several differently expressed circRNAs in TNBC tissues (*N* = 8) compared to non-tumor breast tissues (*N* = 3), and the results from the heat map showed that has_circRNA_101555 (circ-CSNK1G1) was notably upregulated in TNBC tissues (Fig. 1A). Circ-CSNK1G1 attracted our interests and screened for expression analysis. As a result, circ-CSNK1G1 was also highly expressed in tumor tissues (*N* = 33) compared to paired non-cancer tissues (*N* = 33) (Fig. 1B). Next, we measured the expression of circ-CSNK1G1 in some TNBC cell lines. Compared with its expression in MCF-10 A cells, circ-CSNK1G1 expression was significantly increased in MDA-MB-453, MDA-MB-468, MDA-MB-231 and BT-549 cells (Fig. 1C). Combined with the overall survival of these recruited patients with TNBC, we realized that high expression of circ-CSNK1G1 was associated with lower overall survival within 5 years (Fig. 1D), hinting that circ-CSNK1G1 was a risk factor in TNBC development. RNase R treatment showed that circ-CSNK1G1 was resistant to RNase R digestion relative to linear CSNK1G1 in MDA-MB-231 and BT-549 cells (Fig. 1E and F). Moreover, we discovered that circ-CSNK1G1 was mainly expressed in the cytoplasm but not in the nucleus (Fig. 1G and H). These data suggested that circ-CSNK1G1 might participate in TNBC progression.

### Circ-CSNK1G1 interference blocked proliferation, migration, invasion and glycolysis metabolism of TNBC cells

**Fig. 2:**
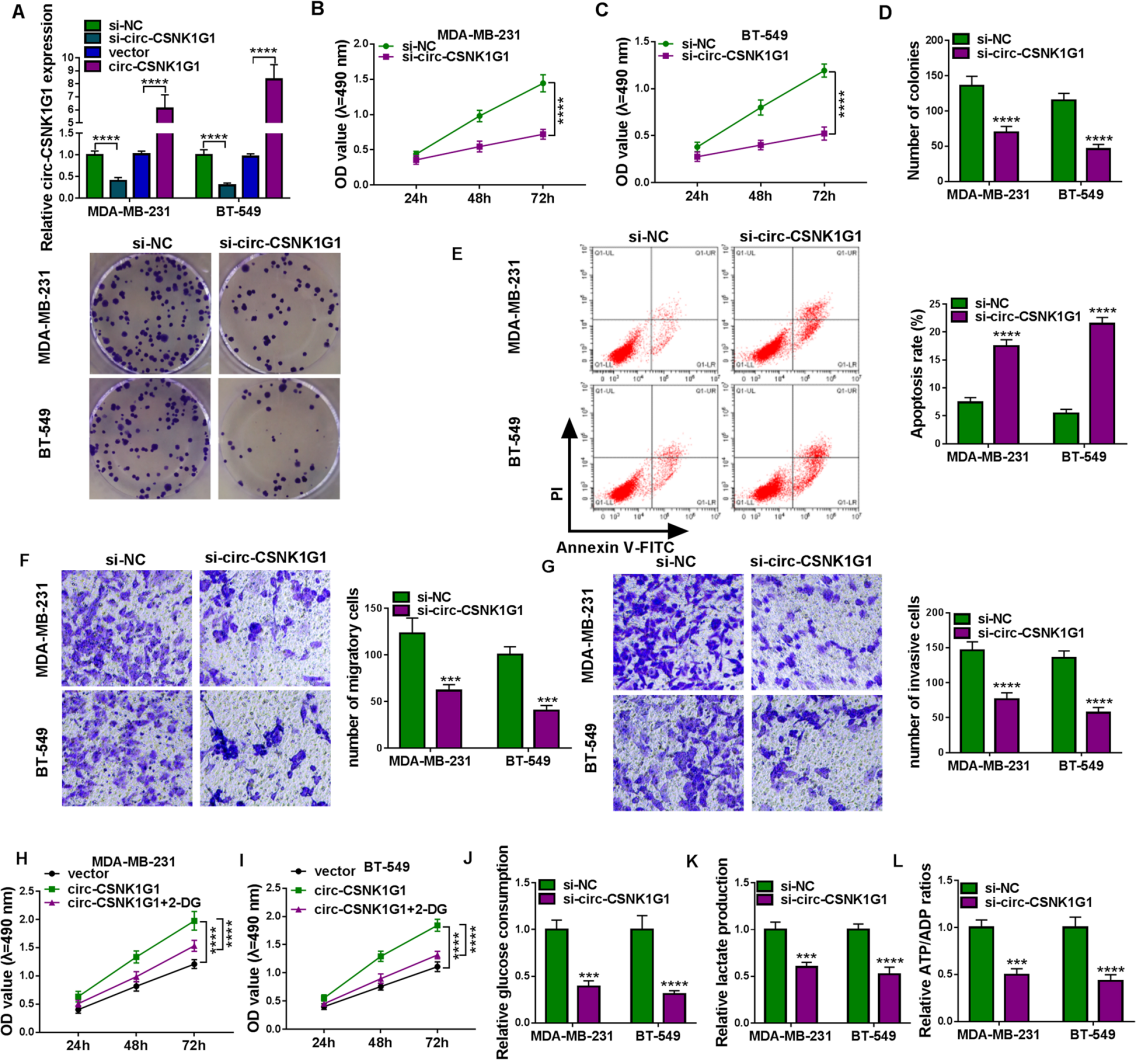
Circ-CSNK1G1 silence inhibited cell proliferation, migration, invasion and glycolysis. **A** The efficiency of circ-CSNK1G1 silence and overexpression was examined by qPCR. **B** and **C** MTT assay was conducted to assess proliferation in MDA-MB-231 and BT-549 cells after circ-CSNK1G1 silence. **D** Colony formation assay was used to assess proliferation in cells after circ-CSNK1G1 silence. **E** Flow cytometry assay was performed to monitor cell apoptosis in cells after circ-CSNK1G1 silence. **F** and **G** Transwell assay was performed to monitor cell migration and invasion in cells after circ-CSNK1G1 silence. **H** and **I** The effects of 2-DG on the role of circ-CSNK1G1 were determined by MTT assay. **J**-**L** Glucose consumption, lactate production and ATP/ADP ratios were detected to assess glycolysis progression. ***P < 0.001 and ****P < 0.0001

Gain-function and loss-function assays were conducted to determine the function of circ-CSNK1G1 in TNBC cells. The efficiency of circ-CSNK1G1 interference and circ-CSNK1G1 overexpression was checked at first, and increased expression of circ-CSNK1G1 in MDA-MB-231 and BT-549 cells transfected with circ-CSNK1G1 and decreased expression of circ-CSNK1G1 in cells transfected with si-circ-CSNK1G1 presented high efficiency of circ-CSNK1G1 overexpression or interference (Fig. 2A). MTT assay indicated that circ-CSNK1G1 knockdown inhibited cell proliferation (Fig. 2B and C), and colony formation assay showed that circ-CSNK1G1 knockdown inhibited colony formation (Fig. 2D). Flow cytometry assay displayed that circ-CSNK1G1 knockdown promoted MDA-MB-231 and BT-549 cell apoptosis (Fig. 2E). Transwell assay pointed out that circ-CSNK1G1 knockdown suppressed cancer cell malignant migration and invasion (Fig. 2 F and G). Besides, circ-CSNK1G1 overexpression markedly promoted cancer cell proliferation, while the proliferation was blocked with the addition of 2-DG, an inhibitor of glycolysis (Fig. 2 H and I). We thus speculated that circ-CSNK1G1 might affect glycolysis energy metabolism in TNBC. Further assays presented the results that circ-CSNK1G1 knockdown resulted in significant decreases of glucose consumption, lactate production and ATP/ADP ration (Fig. 2 J-L), indicating that circ-CSNK1G1 knockdown inhibited glycolysis metabolism. Collectively, circ-CSNK1G1 knockdown suppressed malignant phenotypes in TNBC cells.

### BT-549 cells with circ-CSNK1G1 knockdown led to decreased tumor volume and tumor weight

**Fig. 3:**
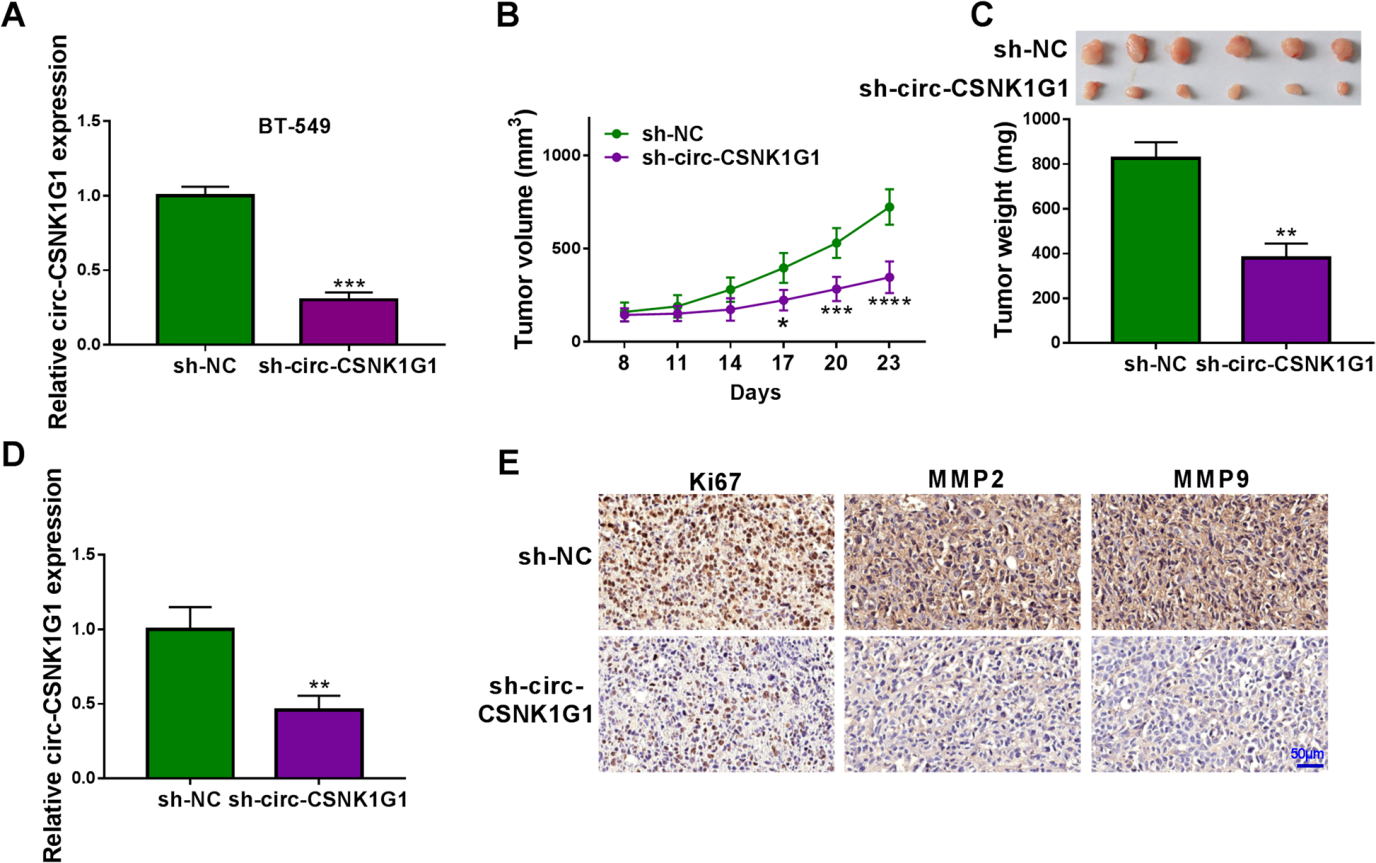
Circ-CSNK1G1 knockdown blocked tumor growth. **A** The expression of circ-CSNK1G1 in BT-549 cells after sh-circ-CSNK1G1 or sh-NC transfection was measured by qPCR. **B** and **C** Tumor volume and tumor weight were measured to monitor tumor growth. **D** The expression of circ-CSNK1G1 in the removed tumor tissues was measured by qPCR. (**E**) The abundance of Ki67, MMP2 and MMP9 in tumor tissues was analyzed by IHC assay. **P < 0.01 and ***P < 0.001

BT-549 cells were infected with sh-circ-CSNK1G1 or sh-NC, and then we found circ-CSNK1G1 expression was notably decreased in BT-549 cells after sh-circ-CSNK1G1 infection (Fig. 3 A). Tumor volume was recorded every three days. The data showed that circ-CSNK1G1 knockdown significantly inhibited tumor volume from day 17 after injection (Fig. 3B). Finally, mice with sh-circ-CSNK1G1 injection harbored poor tumor size and tumor weight compared to sh-NC (Fig. 3C). Moreover, the expression of circ-CSNK1G1 in sh-circ-CSNK1G1-injected tumor tissues was notably lower than that in sh-NC-injected tumor tissues (Fig. 3D). IHC assay presented that the expression of Ki67, MMP2 and MMP9 was strikingly lower in sh-circ-CSNK1G1-administered tumor tissues relative to sh-NC (Fig. 3E), suggesting the growth of tumor tissues was suppressed by sh-circ-CSNK1G1. In short, circ-CSNK1G1 downregulation blocked breast tumor growth in vivo.

### MiR-28-5p was a target of circ-CSNK1G1

**Fig. 4:**
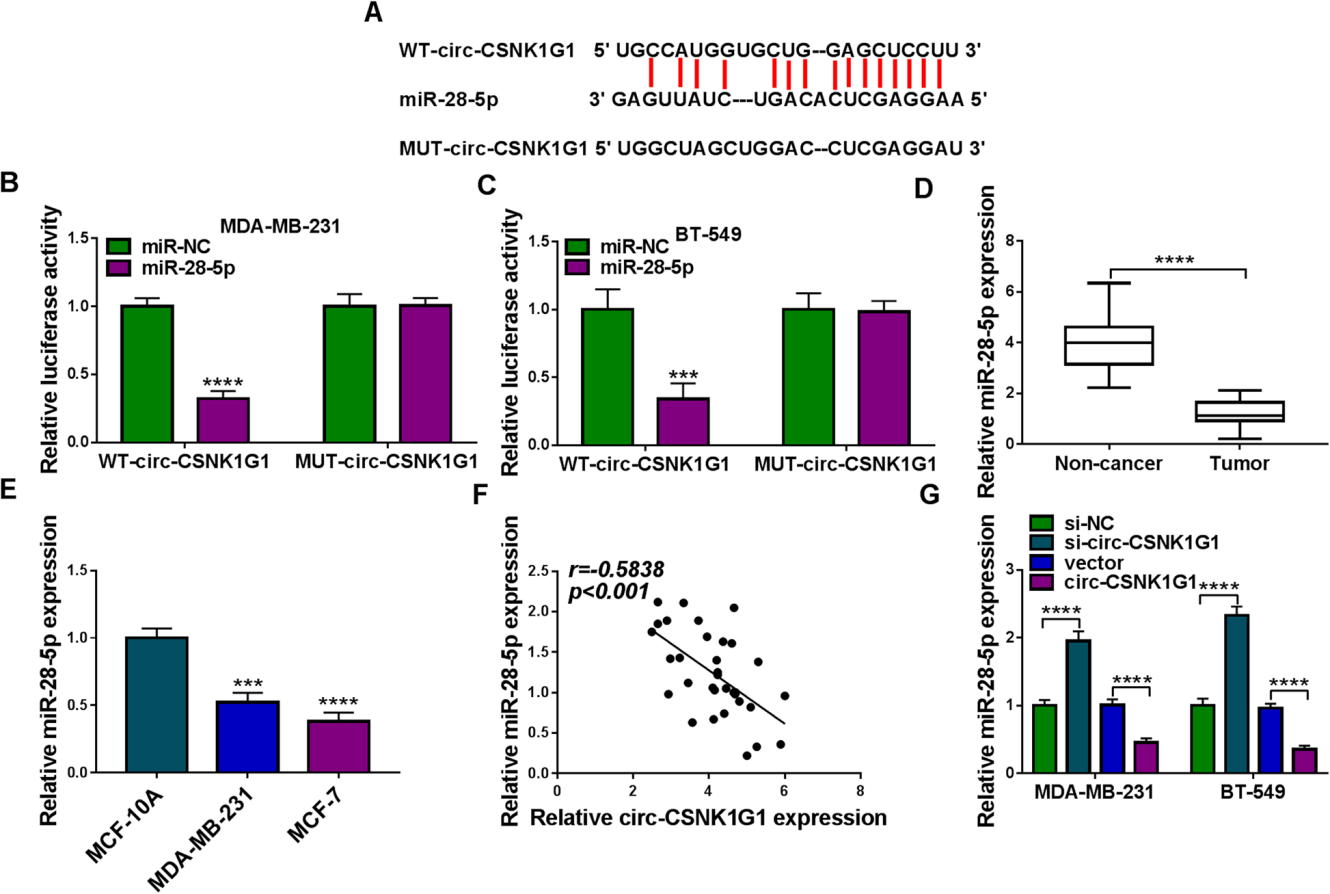
Circ-CSNK1G1 suppressed the expression of miR-28-5p. **A** The binding sites between circ-CSNK1G1 and miR-28-5p. **B** and **C** Luciferase reporter assay was performed to confirm the relationship between circ-CSNK1G1 and miR-28-5p. **D** The expression of miR-28-5p in tumor tissues and normal tissues was detected by qPCR. **E** The expression of miR-28-5p in MCF-10 A, MDA-MB-231 and BT-549 cells was detected by qPCR. **F** The correlation between circ-CSNK1G1 and miR-28-5p expression was analyzed by Pearson analysis. **G** The effects of circ-CSNK1G1 silence and overexpression on miR-28-5p expression were detected by qPCR. ***P < 0.001 and ****P < 0.0001

An online predicting tool starbase v3.0 showed that there were several binding sites between circ-CSNK1G1 sequence and miR-28-5p sequence (Fig. 4A). Dual-luciferase reporter assay was performed using WT and MUT reporter plasmids to validate the relationship between circ-CSNK1G1 and miR-28-5p. The result presented that the cotransfection of miR-28-5p and WT-circ-CSNK1G1 significantly diminished luciferase activities in MDA-MB-231 and BT-549 cells (Fig. 4B and C), indicating that miR-28-5p was a target of circ-CSNK1G1. In addition, the expression of miR-28-5p was notably lower in tumor tissues than that in non-cancer tissues (Fig. 4D). The expression of miR-28-5p was also decreased in MDA-MB-231 and BT-549 cells than that in MCF-10 A cells (Fig. 4E). Besides, miR-28-5p expression in tumor tissues was negatively correlated with circ-CSNK1G1 expression (Fig. 4 F). As expected, the expression of miR-28-5p was enhanced in MDA-MB-231 and BT-549 cells transfected with si-circ-CSNK1G1, while miR-28-5p expression was repressed in cells transfected with circ-CSNK1G1 (Fig. 4G).

### MiR-28-5p inhibition recovered circ-CSNK1G1 knockdown-suppressed TNBC cell proliferation, migration, invasion and glycolysis metabolism

**Fig. 5:**
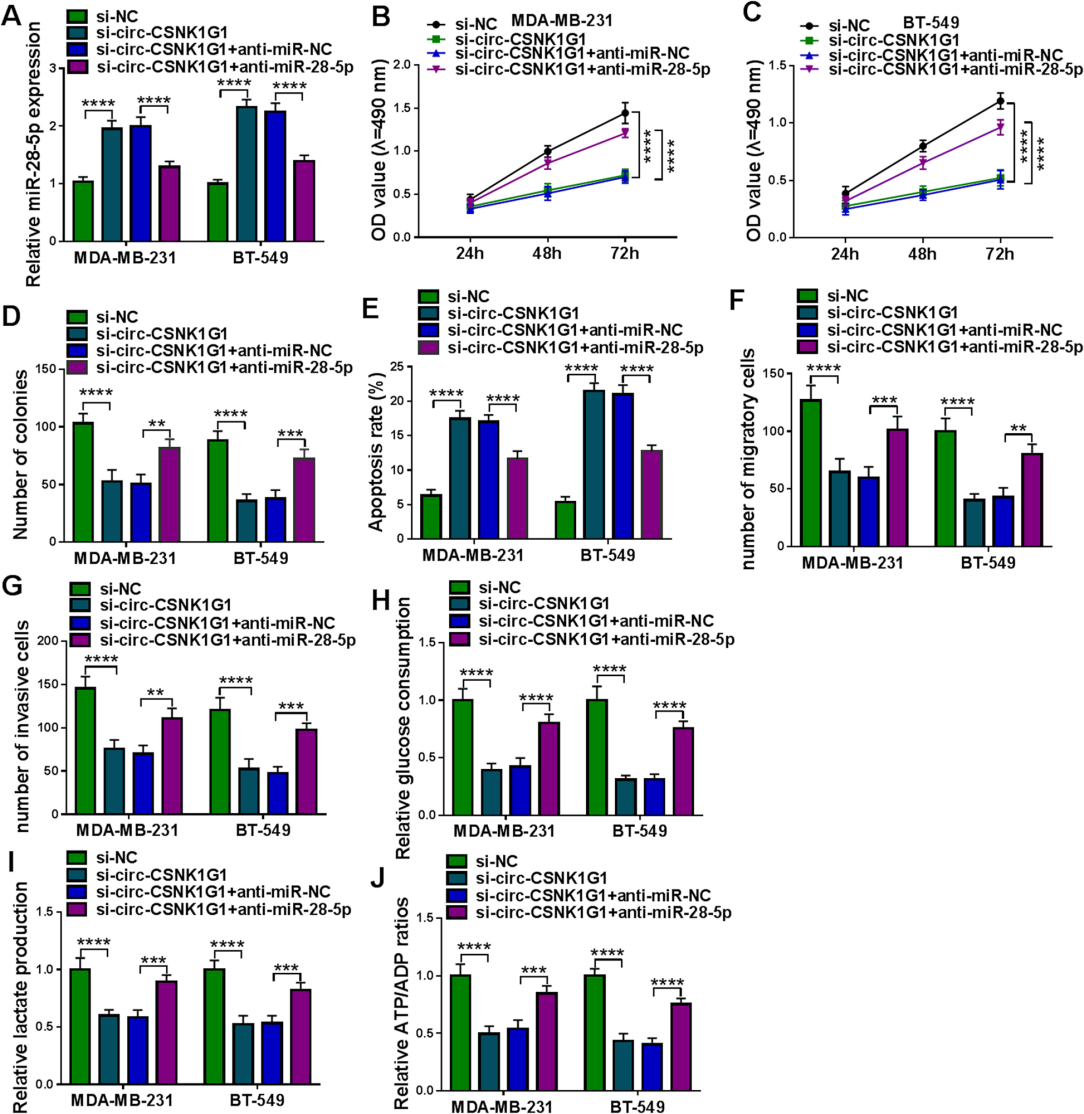
MiR-28-5p deficiency recovered breast cell malignant behaviors suppressed by circ-CSNK1G1 silence. Rescue experiments were performed in MDA-MB-231 and BT-549 cells transfected with si-circ-CSNK1G1 alone or si-circ-CSNK1G1 + anti-miR-28-5p together, using si-NC or si-circ-CSNK1G1 + anti-miR-NC as the control. **A** The expression of miR-28-5p was detected using qPCR. **B**-**D** Cell proliferation was analyzed using MTT assay and colony formation assay. **E** Cell apoptosis was assessed using flow cytometry assay. **F** and **G** Cell migration and cell invasion were investigated using transwell assay. **H**-**J** Glucose consumption, lactate production and ATP/ADP ratios were detected to assess glycolysis progression. **P < 0.01, ***P < 0.001, and ****P < 0.0001

To verify that circ-CSNK1G1 functioned by targeting miR-28-5p, MDA-MB-231 and BT-549 cells were introduced with si-circ-CSNK1G1 or si-circ-CSNK1G1 + anti-miR-28-5p. The expression of miR-28-5p was strikingly increased in cells after si-circ-CSNK1G1 transfection alone but decreased in cells after si-circ-CSNK1G1 + anti-miR-28-5p cotransfection (Fig. 5 A). MTT assay showed that the reintroduction of anti-miR-28-5p promoted MDA-MB-231 and BT-549 cell proliferation compared to si-circ-CSNK1G1 transfection alone (Fig. 5B and C), which was confirmed by colony formation assay (Fig. 5D). Flow cytometry assay disclosed that the cotransfection of si-circ-CSNK1G1 + anti-miR-28-5p inhibited cell apoptosis rate that was induced by si-circ-CSNK1G1 transfection alone (Fig. 5E). Transwell assay discovered that the cotransfection of si-circ-CSNK1G1 + anti-miR-28-5p recovered cell migration and cell invasion that were blocked by si-circ-CSNK1G1 transfection alone (Fig. 5 F and G). In addition, the decreased glucose consumption, lactate production and ATP/ADP ration in MDA-MB-231 and BT-549 cell transfected with si-circ-CSNK1G1 were largely recovered in cells transfected with si-circ-CSNK1G1 + anti-miR-28-5p (Fig. 5 H-J). These data suggested that circ-CSNK1G1 regulated MDA-MB-231 and BT-549 cell proliferation, migration, invasion and glycolysis metabolism by targeting miR-28-5p.

### LDHA was a downstream target of miR-28-5p

**Fig. 6:**
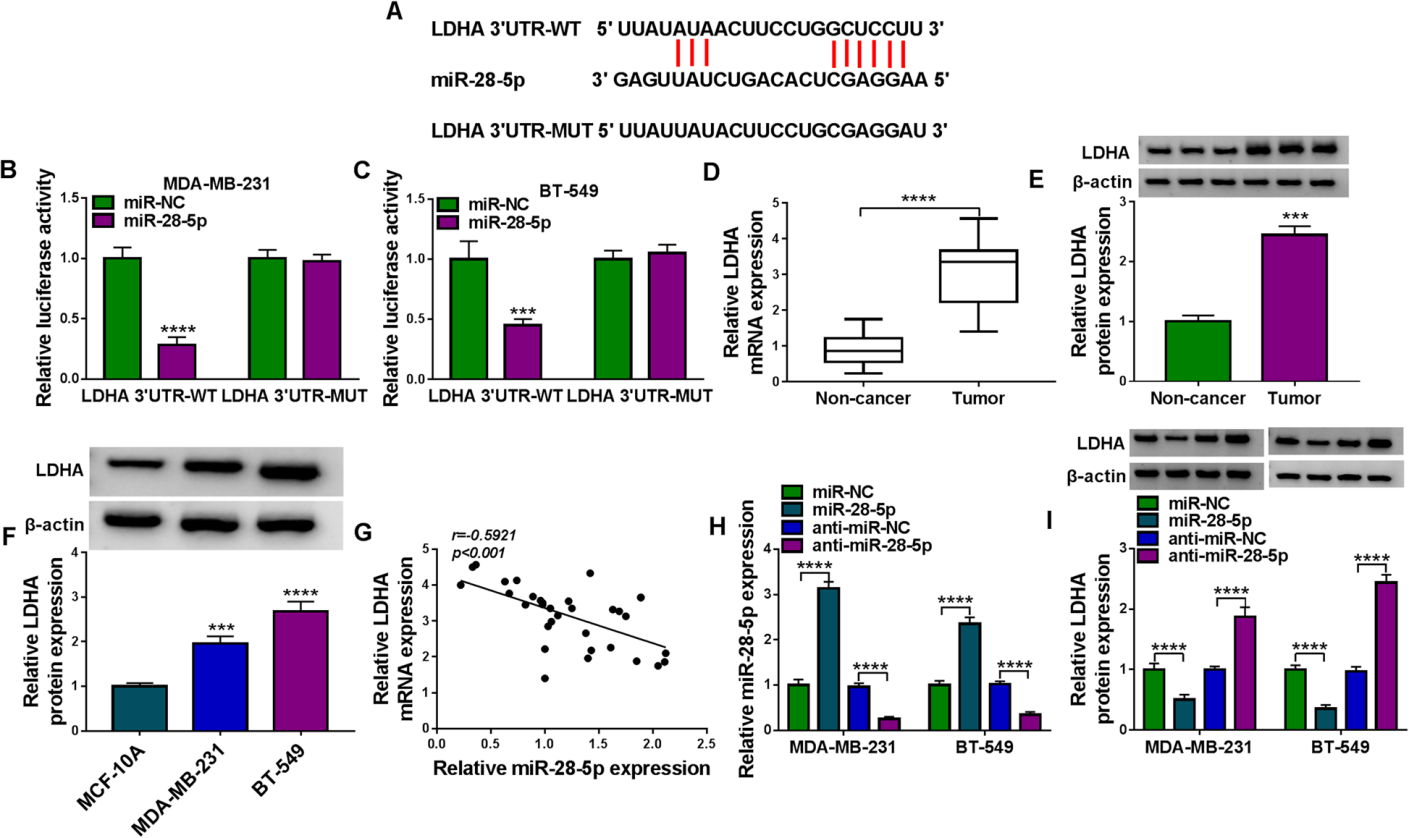
MiR-28-5p suppressed the expression of LDHA. **A** The binding sites between miR-28-5p and LDHA 3’UTR. **B** and **C** Dual-luciferase reporter assay was performed to confirm the interaction between miR-28-5p and LDHA. **D** and **E** The expression of LDHA mRNA and protein in tumor tissues and normal tissues was measured by qPCR and western blot. **F** The expression of LDHA protein in MCF-10 A, MDA-MB-231 and BT-549 cells was measured by western blot. **G** The correlation between LDHA mRNA expression and miR-28-5p expression was analyzed by Pearson analysis. **H** and **I** The effects of miR-28-5p overexpression and deficiency on the expression of LDHA mRNA and protein were determined by qPCR and western blot. ***P < 0.001 and ****P < 0.0001

An online predicting tool starbase v3.0 displayed that miR-28-5p sequence included several binding sites with LDHA 3’UTR (Fig. 6A). Dual-luciferase reporter assay verified their interplays and presented that the cotransfection of miR-28-5p and LDHA 3’UTR-WT could significantly lessen luciferase activity (Fig. 6B C), indicating that LDHA was a target of miR-28-5p. Besides, the expression of LDHA mRNA and LDHA protein was notably promoted in breast tumor tissues compared with that in non-cancer tissues (Fig. 6D and E). The expression of LDHA protein was strikingly enhanced in MDA-MB-231 and BT-549 cells relative to MCF-10 A cells (Fig. 6F). Moreover, LDHA mRNA expression in tumor tissues was negatively correlated with miR-28-5p expression (Fig. 6G). The expression of miR-28-5p was strikingly enhanced in MDA-MB-231 and BT-549 cells transfected with miR-28-5p but repressed in cells transfected with anti-miR-28-5p (Fig. 6 H). On the contrary, the expression of LDHA protein was markedly declined in MDA-MB-231 and BT-549 cells transfected with miR-28-5p but promoted in cells transfected with anti-miR-28-5p (Fig. 6I). The data suggested that LDHA was a target of miR-28-5p.

### LDHA overexpression recovered miR-28-5p-blocked TNBC cell proliferation, migration, invasion and glycolysis metabolism

**Fig. 7:**
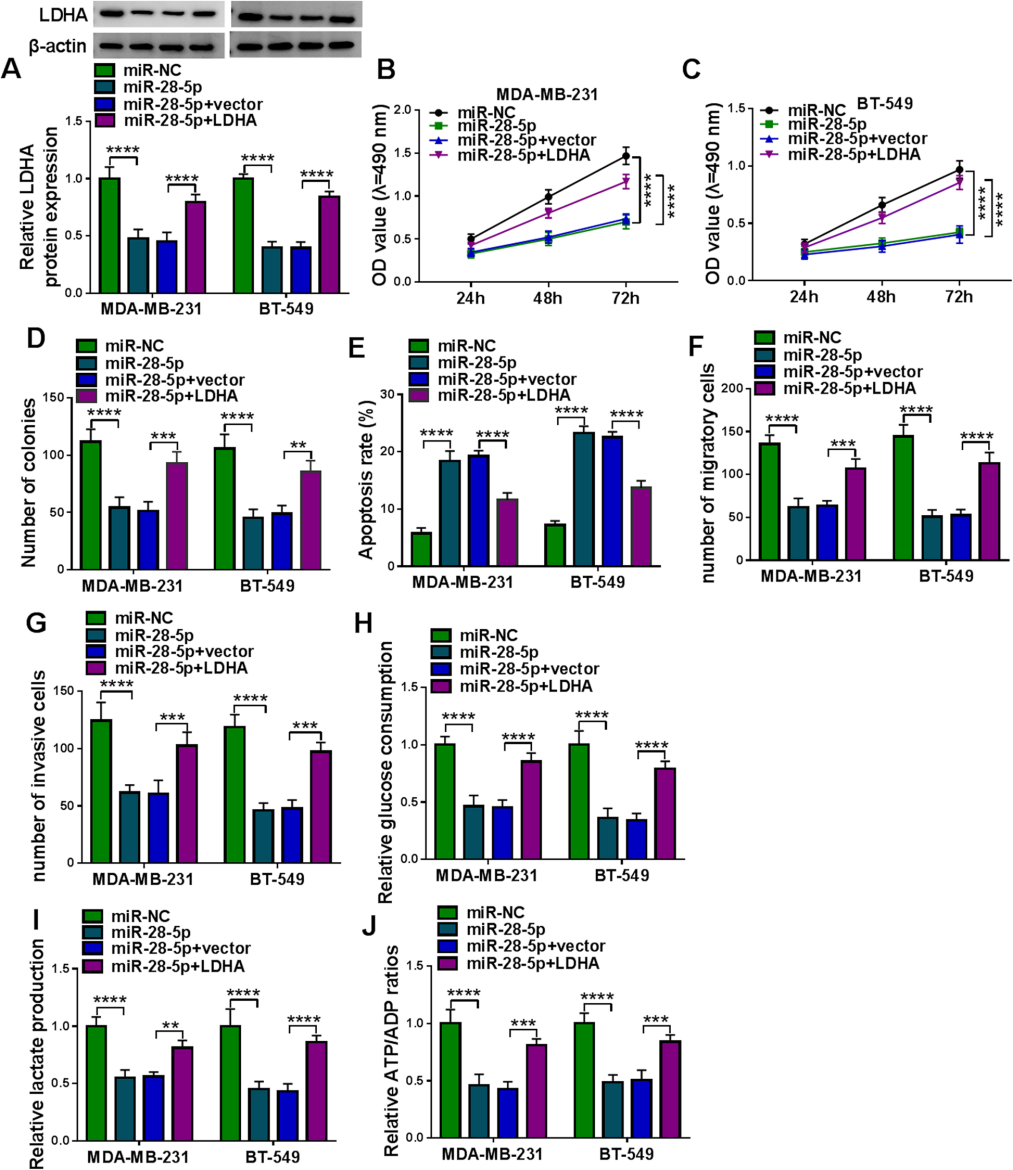
LDHA overexpression recovered TNBC cell malignant behaviors suppressed by miR-28-5p restoration. Rescue experiments were performed in MDA-MB-231 and BT-549 cells transfected with miR-28-5p alone or miR-28-5p + LDHA together, using miR-NC or miR-28-5p + vector as the control. **A** The expression of LDHA protein in these transfected cells was detected by western blot. **B**-**D** Cell proliferation was assessed using MTT assay and colony formation assay. **E** Cell apoptosis was monitored using flow cytometry assay. (**F** and **G**) Cell migration and cell invasion were determined using transwell assay. **H**-**J** Glycolysis metabolism progression was assessed by detecting glucose consumption, lactate production and ATP/ADP ratios. **P < 0.01, ***P < 0.001, and ****P < 0.0001

To confirm that miR-28-5p played functions by inhibiting LDHA, MDA-MB-231 and BT-549 cells were transfected with miR-28-5p or miR-28-5p + LDHA. The expression of LDHA protein was notably decreased in MDA-MB-231 and BT-549 cells transfected with miR-28-5p, while the expression of LDHA protein was recovered in cells transfected with miR-28-5p + LDHA (Fig. 7A). MTT assay showed that miR-28-5p transfection markedly suppressed cell proliferation, while miR-28-5p + LDHA transfection markedly recovered cell proliferation (Fig. 7B C), which was also verified by colony formation assay (Fig. 7D). Flow cytometry assay manifested that miR-28-5p overexpression significantly promoted cell apoptosis, while the reintroduction of LDHA alleviated cell apoptosis (Fig. 7E). Transwell assay indicated that cell migration and cell invasion were blocked by miR-28-5p overexpression but largely recovered by the reintroduction of LDHA (Fig. 7F and G). In addition, LDHA overexpression recovered the levels of glucose consumption, lactate production and ATP/ADP radio that were weakened by miR-28-5p restoration (Fig. 7 H-J). The data suggested that miR-28-5p inhibited TNBC malignant development by suppressing LDHA.

### The expression of LDHA weakened by circ-CSNK1G1 knockdown was enhanced by mir-28-5p inhibition

**Fig. 8:**
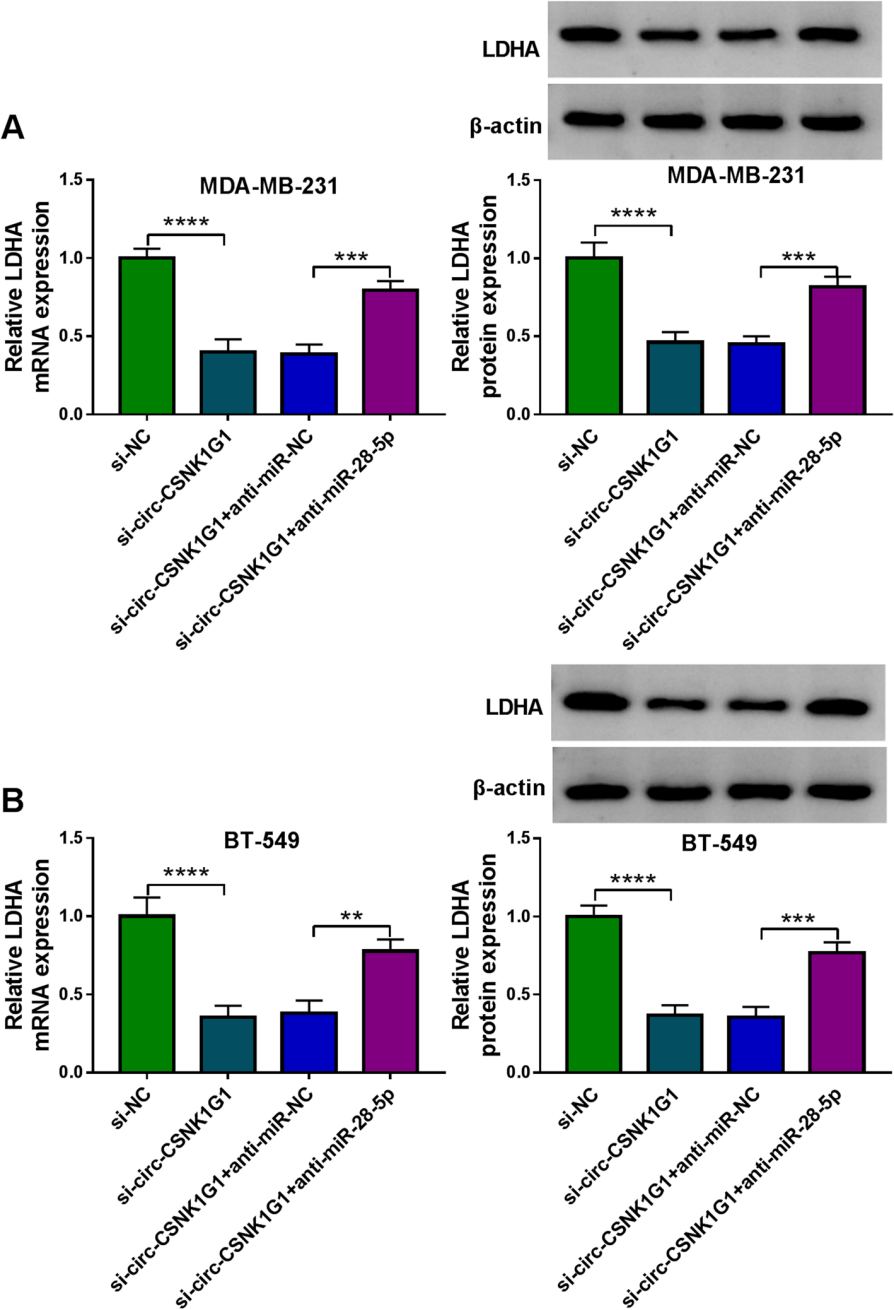
Circ-CSNK1G1 decoyed miR-28-5p to release LDHA. **A** and **B** The effects of circ-CSNK1G1 knockdown alone or circ-CSNK1G1 knockdown combined with miR-28-5p inhibition on the expression of LDHA was detected using qPCR and western blot. **P < 0.01, ***P < 0.001, and ****P < 0.0001

The expression of LDHA mRNA and protein was pronouncedly decreased in MDA-MB-231 and BT-549 cells transfected with si-circ-CSNK1G1 compared to si-NC, while the expression of LDHA mRNA and protein was pronouncedly recovered in MDA-MB-231 and BT-549 cells transfected with si-circ-CSNK1G1 + anti-miR-28-5p compared to si-circ-CSNK1G1 + anti-miR-NC (Fig. 8 A and 8B). The data suggested that circ-CSNK1G1 regulated the expression of LDHA by suppressing miR-28-5p.

## Discussion

Though different circRNA expression profiles summarized numerous circRNAs that were upregulated or downregulated in breast cancer [14, 19, 20], the functions of most circRNA are still unknown. Our study devoted to the exploration of the function and action mechanism of circ-CSNK1G1 in TNBC. The findings mainly manifested that circ-CSNK1G1 expression was abnormally enhanced in tumor tissues and cancer cells. Functional assays showed that circ-CSNK1G1 knockdown notably blocked the development of TNBC, which was attributed to the role of circ-CSNK1G1-mediated miR-28-5p/LDHA pathway. Our study not only determined the role of circ-CSNK1G1 in TNBC but also addressed a potential mechanism for its function.

Circ-CSNK1G1 was firstly identified to be overexpressed in breast cancer tissues in a circRNA profile [14]. The similar expression pattern of circ-CSNK1G1 in breast cancer was also revealed in another microarray data [21], highlighting the dysregulation of circ-CSNK1G1 in breast cancer. Besides, circ-CSNK1G1 was also demonstrated to be highly expressed in hepatocellular carcinoma (HCC) tissues, and circ-CSNK1G1 knockdown inhibited HCC cell proliferation and colony formation, as well as tumor growth in vivo [22]. Interestingly, the expression of circ-CSNK1G1 was also shown to be increased in colorectal cancer [23]. Consistent with these findings, our data observed a significant increase of circ-CSNK1G1 expression in TNBC tissues and cells. Innovatively, our study explored the detailed function of circ-CSNK1G1 and discovered that circ-CSNK1G1 knockdown inhibited cancer cell proliferation, colony formation, migration, invasion and glycolysis energy metabolism in vitro and blocked tumor formation and growth in vivo. We thus defined that circ-CSNK1G1 was a cancer-driver in TNBC.

To illustrate the functional mechanism of circ-CSNK1G1, we screened and verified the target miRNAs of circ-CSNK1G1. As a result, miR-28-5p was ensured as a target of circ-CSNK1G1. Previous studies summarized that miR-28-5p acted as a tumor suppressor to block the development of various cancers, such as HCC and colorectal cancer [24, 25]. Intriguingly, a recent study disclosed the anti-tumor role of miR-28-5p in breast cancer, and the data suggested that miR-28-5p deficiency largely recovered breast cancer cell proliferation, migration and invasion that were repressed by long non-coding RNA MCM3AP-AS1 [26]. MiR-28-5p deficiency in our study promoted the inhibitory effects on cell proliferation, colony formation, migration, invasion and glycolysis energy metabolism caused by circ-CSNK1G1 knockdown, while miR-28-5p restoration inhibited these malignant behaviors.

MiRNAs regulate gene expression by interacting with their 3’UTR. MiR-28-5p bound to LDHA 3’UTR, and their interaction was further confirmed by dual-luciferase reporter assay. Previous studies claimed that the expression level of LDHA was increased in breast cancer, and LDHA expression was strikingly correlated with TNM stage and distant metastasis [18]. Besides, LDHA was frequently reported to be involved in glucose metabolism. For instance, LDHA overexpression reinforced cell proliferation and glycolysis in breast cancer cells [27]. LDHA-induced glycolysis energy metabolism was suppressed by miR-30a-5p, thus breast tumor growth and metastasis were blocked [28]. Similarly, LDHA overexpression in our study abolished the effects of miR-28a-5p restoration and then recovered cell proliferation, migration, invasion and glycolysis in TNBC.

Our study is a preliminary study to exploit the role of circ-CSNK1G1 in TNBC. Our study, to a certain degree, discloses the functional effects and mechanism of circ-CSNK1G1 in TNBC, which elucidates the pathogenesis of TNBC from the perspective of circRNA dysregulation. Of note, we only study one miRNA/mRNA pathway targeted by circ-CSNK1G1. There are numerous putative miRNAs and downstream mRNAs in the circ-CSNK1G1-regualted networks in TNBC, which should be further explored in future work.

Collectively, this study was the first to investigate the function of circ-CSNK1G1 in TNBC. We mainly found that circ-CSNK1G1 acted as a cancer-driver to promote cell proliferation, migration, invasion and glycolysis energy metabolism in TNBC through the miR-28-5p/LDHA pathway. Our study deepens the understanding of TNBC pathogenesis and risk factors.

Abbreviations.

Circular RNAs (circRNAs);triple-negative breast cancer (TNBC); Quantitative real-time polymerase chain reaction (qPCR); 2-DeoxyGlucose (2-DG); short hairpin RNA (shRNA); Immunohistochemical (IHC); wild-type (WT) and mutant (MUT); analysis of variance (ANOVA);

## Supplementary Information

**Additional file 1.**

## Data Availability

The analyzed data sets generated during the present study are available from the corresponding author on reasonable request.

## References

[1] Siegel RL, Miller KD, Jemal A (2019). Cancer statistics, 2019. CA Cancer J Clin.

[2] Pasculli B, Barbano R, Parrella P (2018). Epigenetics of breast cancer: Biology and clinical implication in the era of precision medicine. Semin Cancer Biol.

[3] Mathe A (2015). Novel genes associated with lymph node metastasis in triple negative breast cancer. Sci Rep.

[4] Nicolini A, Ferrari P, Duffy MJ (2018). Prognostic and predictive biomarkers in breast cancer: Past, present and future. Semin Cancer Biol.

[5] Wang X, Fang L (2018). Advances in circular RNAs and their roles in breast Cancer. J Exp Clin Cancer Res.

[6] Zhang X (2019). Parallel Analyses of Somatic Mutations in Plasma Circulating Tumor DNA (ctDNA) and Matched Tumor Tissues in Early-Stage Breast Cancer. Clin Cancer Res.

[7] Bao-Caamano A, Rodriguez-Casanova A, Diaz-Lagares A (2020). Epigenetics of Circulating Tumor Cells in Breast Cancer. Adv Exp Med Biol.

[8] Vitale SG et al. Peroxisome Proliferator-Activated Receptor Modulation during Metabolic Diseases and Cancers: Master and Minions. PPAR Res. 2016;2016:6517313. 10.1155/2016/6517313 PMC5225385 28115924

[9] Pei X (2020). Circular RNA circ-ZEB1 acts as an oncogene in triple negative breast cancer via sponging miR-448. Int J Biochem Cell Biol.

[10] He D et al. The Novel Circular RNA Circ-PGAP3 Promotes the Proliferation and Invasion of Triple Negative Breast Cancer by Regulating the miR-330-3p/Myc Axis. Onco Targets Ther. 2020;13:10149–10159. 10.2147/OTT.S274574 PMC7553664 33116597

[11] Yang R (2019). The circRNA circAGFG1 acts as a sponge of miR-195-5p to promote triple-negative breast cancer progression through regulating CCNE1 expression. Mol Cancer.

[12] Ebbesen KK, Hansen TB, Kjems J (2017). Insights into circular RNA biology. RNA Biol.

[13] Hsiao KY, Sun HS, Tsai SJ (2017). Circular RNA - New member of noncoding RNA with novel functions. Exp Biol Med (Maywood).

[14] Xu JZ (2019). circTADA2As suppress breast cancer progression and metastasis via targeting miR-203a-3p/SOCS3 axis. Cell Death Dis.

[15] Xin Z (2017). The understanding of circular RNAs as special triggers in carcinogenesis. Brief Funct Genomics.

[16] Matoulkova E (2012). The role of the 3’ untranslated region in post-transcriptional regulation of protein expression in mammalian cells. RNA Biol.

[17] Bagheri F (2016). Tumor-promoting function of single nucleotide polymorphism rs1836724 (C3388T) alters multiple potential legitimate microRNA binding sites at the 3’-untranslated region of ErbB4 in breast cancer. Mol Med Rep.

[18] Huang X (2016). High expressions of LDHA and AMPK as prognostic biomarkers for breast cancer. Breast.

[19] Li Z et al. Profiling and integrated analysis of differentially expressed circRNAs as novel biomarkers for breast cancer. J Cell Physiol. 2020;235(11):7945–59. 10.1002/jcp.29449 31943203

[20] Fu B (2018). Circular RNA profile of breast cancer brain metastasis: identification of potential biomarkers and therapeutic targets. Epigenomics.

[21] Afzali F, Salimi M (2019). Unearthing Regulatory Axes of Breast Cancer circRNAs Networks to Find Novel Targets and Fathom Pivotal Mechanisms. Interdiscip Sci.

[22] Yao Z (2019). Circ_0001955 facilitates hepatocellular carcinoma (HCC) tumorigenesis by sponging miR-516a-5p to release TRAF6 and MAPK11. Cell Death Dis.

[23] Ding B (2020). Whole-transcriptome analysis reveals a potential hsa_circ_0001955/hsa_circ_0000977-mediated miRNA-mRNA regulatory sub-network in colorectal cancer. Aging.

[24] Zhou SL (2016). miR-28-5p-IL-34-macrophage feedback loop modulates hepatocellular carcinoma metastasis. Hepatology.

[25] Almeida MI (2012). Strand-specific miR-28-5p and miR-28-3p have distinct effects in colorectal cancer cells. Gastroenterology.

[26] Chen Q (2020). LncRNA MCM3AP-AS1 promotes breast cancer progression via modulating miR-28-5p/CENPF axis. Biomed Pharmacother.

[27] Xiao X (2016). The miR-34a-LDHA axis regulates glucose metabolism and tumor growth in breast cancer. Sci Rep.

[28] Li L (2017). miR-30a-5p suppresses breast tumor growth and metastasis through inhibition of LDHA-mediated Warburg effect. Cancer Lett.

